# AM fungi modulate organic nitrogen preference of *Eucalyptus* to enhance seedling growth under low fertilization

**DOI:** 10.3389/fpls.2025.1597451

**Published:** 2025-06-20

**Authors:** Xinyue Li, Chunyu Huo, Xinzhu Yang, Qiutong Liu, Zijia Su, Xinyi Fan, Zhihong Liu, Zujing Chen

**Affiliations:** ^1^ Guangdong Key Laboratory for Innovative Development and Utilization of Forest Plant Germplasm, College of Forestry and Landscape Architecture, South China Agricultural University, Guangzhou, China; ^2^ State Key Laboratory of Conservation and Utilization of Subtropical Agro-Bioresources, Guangdong Laboratory for Lingnan Modern Agriculture, South China Agricultural University, Guangzhou, China

**Keywords:** arbuscular mycorrhiza, organic nitrogen, nitrogen metabolism, phosphorus, fertilization, *Eucalyptus*

## Abstract

**Introduction:**

*Eucalyptus* is one of the most productive trees, with short-rotation and high values. Fertilization and inoculating arbuscular mycorrhizal (AM) fungi can increase *Eucalyptus* productivity. Many studies have concentrated on plant absorption of inorganic nitrogen, but less on organic nitrogen and the effect of AM fungi on organic nitrogen acquisition.

**Methods:**

In this study, we set 28 treatments including with or without *Rhizophagus irregularis* inoculation, three organic nitrogen sources (glycine, L-glutamine and peptone) and four levels of nitrogen and phosphorus fertilization, with no application of organic nitrogen fertilizer as control.

**Results and Discussion:**

The results indicated that the height, ground diameter and crown width of *Eucalyptus* seedlings under 2.5 mM glycine and 0.3 mM Pi (G2P2) treatment were 3.23, 0.82 and 3.70 times higher than those without organic nitrogen under 0.03 mM Pi (N0P1) treatment, respectively. Inoculation with AM fungi contributed 10.85%-162.00% increase in dry weight compared to non-mycorrhizal (NM) treatments. Mycorrhizal seedlings also showed significantly higher total phosphorus contents, nitrogen use efficiency and activity of glutamine synthetase, relative to NM treatments. The relative expressions of genes related to organic nitrogen transport (*EgAAP3*, *EgLHT1*, *EgProt2*) and nitrate nitrogen transport (*EgNPF4.5*, *EgNPF6.3*, *EgNPF8.1*) were up-regulated in mycorrhizal *Eucalyptus* roots, compared to the NM treatment. Non-mycorrhizal *Eucalyptus* favored peptone, while mycorrhizal *Eucalyptus* preferred glycine. *Eucalyptus* growth under 2.5 mM organic nitrogen and 0.3 mM Pi (N2P2) treatments were promoted markedly, regardless of organic nitrogen source. Whereas, the growth of mycorrhizal *Eucalyptus* under 0.25 mM organic nitrogen and 0.3 mM Pi (N1P2) treatments were increased the most. This study explored the organic nitrogen and AM fungi fertilizer mode for *Eucalyptus*, this has potential to improve production practice of *Eucalyptus* under low nitrogen and phosphorus condition.

## 1 Introduction

*Eucalyptus* is one of the most widely planted broad-leaf forest species in the world. This tree is characterized by rapid growth, high timber yield, stress resistance, economic value, tolerance to infertile soil, salinity and moisture as well as ideal trunk shape for timber production (Xie et al., 2017; Zhang and Wang, 2021; Shang et al., 2024). However, successive planting of *Eucalyptus* and its strong abilities to absorb water and nutrients cause the soil fertility decline significantly (Cui et al., 2023; Tang et al., 2023). Therefore, fertilization plays a vital role on sustainable development and enhancing *Eucalyptus* timber yield (Yu et al., 2019; Wang et al., 2024).

Nitrogen (N) and phosphorus (P) are the most important and common limiting elements that participate in numerous growth and metabolic processes in plant tissues, thereby determine plants productivity (Marro et al., 2022; Rengel et al., 2022; Yahaya et al., 2023). The two fertilizers are coordinated and interact with each other during crop uptake and utilization (Kumar et al., 2021). P and N-related signaling pathways can be categorized into three classes: phosphate starvation response, nitrogen starvation response and primary nitrate response (Bouain et al., 2019; Krouk and Kiba, 2020; Zhang et al., 2024). Under typical conditions, N application enhances P uptake and utilization by increasing soil phosphatase activity, which accelerates P conversion in the soil (Hu et al., 2023). This mutual promotion improves soil fertility and reduces fertilizer waste. Consequently, the combined application of N and P fertilizers is more effective than the single application of either fertilizer (Zheng et al., 2023). However, excessive reliance on inorganic fertilizers can exacerbate soil degradation and environmental pollution. In low-fertility soils, traditional fertilization strategies often fail to address the complex nutrient limitations faced by *Eucalyptus*, highlighting the need for alternative approaches to improve nutrient use efficiency and sustainability.

Arbuscular mycorrhizal (AM) fungi are soil microorganisms that form symbiotic relationships with most terrestrial plants (Duan et al., 2024). Previous studies showed that mycorrhizal fungi induce plants to produce various phytohormones and growth-regulating substances (Pons et al., 2020; Ahmed et al., 2025). They also stimulate the production of antimicrobial compounds, activate defense-related enzymes, and regulate plant physiological metabolism (Weng et al., 2022; Wahab et al., 2023). These actions promote healthy growth and enhance disease resistance. AM fungi can extract substantial amounts of N from organic matter (Vaishnav et al., 2025). They may play a previously unappreciated role in N cycle by intercepting inorganic N released from decomposing organic matter before it reaches the root system, transferring some to the plant (Hodge and Fitter, 2010). AM fungi influence the soil N cycle (Fall et al., 2022). AM fungi also enhance plant growth and nutrition by acquiring more N, P, and other less mobile nutrients (Saia et al., 2019; Kuila and Ghosh, 2022). At the molecular level, AM fungi regulate the expression of phosphate transporter protein genes (Loth-Pereda et al., 2011; Duan et al., 2024). This enhances effective P uptake and transport processes in response to environmental P fluctuations (Navarro and Morte, 2024). This symbiotic relationship not only enhances nutrient acquisition, but also reduces fertilizer dependency, making it a promising strategy for sustainable forestry (Xie K, et al., 2022; Sun K, et al., 2024). Recently, it was showed the strongest effect of AM fungi inoculation under low fertilization (Sun J, et al., 2024; Mbodj et al., 2025). Under low fertilization, AM fungi inoculation reduced yield losses and resulted in higher yields.

Most N in soil exists mainly as insoluble polymeric N-containing compounds. In natural ecosystems, symbionts predominantly utilize organic N through the uptake of soluble organic N sources. These insoluble macromolecular organic N compounds are absorbed and utilized by host plants, mycorrhizae, and mycorrhizal fungal hyphae when they are enzymatically broken down into soluble small molecule (Jones et al., 2005; Wahab et al., 2024). Previous study has found that organic fertilization can improve soil aggregation by increasing the products of AM fungi (Řezáčová et al., 2021). AM fungi can fix N from organic sources, while organic N is not transferred to the plant in its intact form (Hodge et al., 2001; Hodge and Fitter, 2010; Jansa et al., 2019). AM fungi acquired approximately 1/3 of the N from degraded organic N matter and transferred 3% of this content to plants (Hodge and Fitter, 2010). The quantum dots labeling method was used to demonstrate that AM fungi can directly uptake and transport glycine, and quantum dots-labeled glycine was present in soil extra-radical hyphae, plant roots and plant stems (Whiteside et al., 2009, 2012). Besides, AM fungi assimilate NH_4_^+^ mainly through the glutamine synthetase-glutamate synthetase (GS-GOGAT) pathway, with glutamine and glutamate being abundant in AM fungi mycelium (Wu XL, et al., 2024). Root uptake of amino acids involves transporters such as lysine histidine transporter 1 (*LHT1*), which plays a key role in organic N acquisition in model plants like *Arabidopsis thaliana* (Hirner et al., 2006; Svennerstam et al., 2011; Ganeteg et al., 2017). These findings suggest that AM fungi may preferentially utilize organic N sources, offering a distinct advantage in low-fertility soils where inorganic N is scarce.

In recent years, N fertilizer mainly focused on the study of plant uptake of inorganic N and metabolic mechanism. However, there is less research on organic N. In addition, the symbiosis of arbuscular mycorrhiza and plants uptake organic N has not been studied much. This study establishes a symbiosis system between AM fungi and *Eucalyptus*. The purpose is to explore suitable organic N fertilizer and fertilization mode. We hypothesized that (1) mycorrhizal treatments under organic N and P fertilization would markedly promote *Eucalyptus* growth, and (2) mycorrhization would promote *Eucalyptus* organic N metabolism and N use efficiency.

## 2 Materials and methods

### 2.1 Plant material, AM fungi inoculation and experimental design

The pot sample were three-month-old asexual group-cultivated seedlings of *Eucalyptus urophylla* × *Eucalyptus grandis* DH3229 from Gaoyaojiayao forestry development company. The inoculated AM fungi was *Rhizophagus irregularis* DAOM 197198, from the mycorrhizal biology team, College of Forestry and Landscape Architecture, South China Agricultural University. The conditions for the germination of *R. irregularis* were as follows: spores were washed with sterile water several times until the inhibitors were cleaned and stored in the refrigerator at 4°C. The spores were stored in a 12-well cell culture plate containing sterile water and placed in a dark incubator at 25°C to induce the spores to produce germination tubes. After 7 days of cultivation, the germinated spores were collected in centrifuge tubes and used for plant inoculation tests. The host plant was *Zea mays* and the resulting mycorrhizal agent was mixture of AM fungal spores, mycelium, root segments and soil. Spores were collected by wet sieving and sucrose centrifugation (Yamamura et al., 2003). Seedling roots were inoculated with *R. irregularis*, pipetting 1 mL of an aqueous solution containing approximately 400 spores to the vicinity of the root system. Each non-mycorrhizal seedling received the same amount of sterilized autoclaved inoculum (15 min in an autoclave at 121°C). One month after inoculating AM fungi, the root symbiosis of the seedlings was observed under a microscope to confirm successful inoculation, and then samples were collected.

In this study, there were 28 treatments in total, including with or without AM fungi inoculation, three organic N sources (glycine, L-glutamine and peptone) and four levels of N and P fertilization, with no application of organic N fertilizer as control. Four biological replicates were set up for each treatment. The plant growth conditions were: photoperiod of 16 h at 24°C during the day with a light intensity of 100-200 W m^-2^ and 8 h at 19°C at night. Watering with Low (30 µM L^-1^ NaH_2_PO_4_) Long Ashton (mLA) nutrient solution (Hewitt, 1966) during the previous month of mycorrhizal colonization. After mycorrhizal symbiosis, the improved LA nutrient solution with different levels of organic N, N and P was used for watering, with 50 mL of water per *Eucalyptus* seedling every three days. N concentration of 0.25 mM NaNO_3_ was added to the base of organic N (glycine, L-glutamine, and peptone). Two level of organic N concentrations were set- in the study, 0.25 mM and 2.5 mM. Two level of P concentrations were set, 0.03 mM and 0.3 mM. N concentration of 1 mM NaNO_3_ was added as control treatments with no organic N fertilization. The specific treatment of applying organic N and P concentration is shown in the table below (**Table 1**).

**Table 1:** The amounts of organic nitrogen (ON) and phosphorus (P) fertilization.

Nitrogen source	Treatment	ON concentration	P concentration
Control	N0P1	0	0.03 mM
	N0P2	0	0.3 mM
Glycine	G1P1	0.25 mM	0.03 mM
	G1P2	0.25 mM	0.3 mM
	G2P1	2.5 mM	0.03 mM
	G2P2	2.5 mM	0.3 mM
L-glutamine	L1P1	0.25 mM	0.03 mM
	L1P2	0.25 mM	0.3 mM
	L2P1	2.5 mM	0.03 mM
	L2P2	2.5 mM	0.3 mM
Peptone	P1P1	0.25 mM	0.03 mM
	P1P2	0.25 mM	0.3 mM
	P2P1	2.5 mM	0.03 mM
	P2P2	2.5 mM	0.3 mM

The diameter and height of the seedlings were measured monthly from December 2023 to January 2024 using straightedge and vernier calipers. *Eucalyptus* seedling roots and leaves were harvested and weighed freshly, killed at 105°C for 15 min, then dried at 70°C until constant weight and weighed to calculate the biomass. Root length, diameter, surface area and root volume of seedlings were determined using a root scanner (WinRHIZO, China). The aboveground and underground part of each biological replicate was randomly divided into 5 groups for the determination of chlorophyll concentration, photosynthetic gas exchange parameters, enzyme activity, total nitrogen (TN) contents, total phosphorus (TP) contents and gene levels, respectively.

### 2.2 Eucalyptus leaf photosynthetic characteristic measurements

Gas exchange parameters of *Eucalyptus* leaves were determined with Li-6400 portable photosynthesis meter (LI-COR, USA), mainly including net photosynthetic rate, stomatal conductance, transpiration rate and intercellular CO_2_ concentration. The measurements were made on the top 3 unfolded leaves of *Eucalyptus* seedlings between 08:00 to 12:00 am in the morning on a sunny day. Three measurement points were randomly selected for each leaf (Zhang et al., 2018).

For the determination of photosynthetic pigments, the ethanol extraction method was used. 0.2 g leaves were taken into a 50 mL centrifuge tube, and 25 mL of 95% ethanol was added sequentially to the tube and sealed, and then extracted for 24-36 h at room temperature in the dark, and then the extracts were diluted twice, and then colorimetrically compared with the UV-visible spectrophotometer at wavelengths of 665 and 649 nm. The concentrations of chlorophyll a, b and the total chlorophyll were calculated according to the OD values at each wavelength (Lichtenthaler and Wellburn, 1983).

### 2.3 Total N P contents, nitrogen use efficiency and enzyme activity measurements

Dry roots were fully ground and homogenized for nutrient analysis. The total nitrogen (TN) content was measured from 0.2 g of dried roots powder by the Kjeldahl method (Schuman et al., 1973). Nitrogen use efficiency (NUE) was calculated as the dry weights of the seedlings divided by the TN contents of seedlings (Shi et al., 2017).

To measure the total phosphorus (TP) contents, 0.4 g of dried roots powder was digested with HNO_3_. The specimens were determined by inductively coupled plasma mass spectrometry (ICP-MS) after digestion and the internal standard method was used for determination of TP contents (Hansen et al., 2013). The glutamate synthase (GOGAT) activity of *Eucalyptus* in the leaves was determined using the Ionization assay (Singh and Srivastava, 1986). The GOGAT activity was expressed as the reduction of NADH in 0.1 g of sample after ten minutes’ reaction. The nitrate reductase (NR) activity of *Eucalyptus* in the leaves was determined using the ex vivo assay (Srivastava, 1980). The NR activity was expressed as the amount of nitrite produced by 0.15 g of sample after ten minutes’ reaction. The glutamine synthetase (GS) activity of *Eucalyptus* in the leaves was determined using the GS activity assay (Magalhaes and Huber, 1991). The GS activity was expressed as the amount of γ-glutamyl isohydroxycyclohexanoic acid produced by 0.05 g of sample.

### 2.4 Determination of nitrogen relative gene expression levels

Real-time fluorescence quantitative PCR (qRT-PCR) was used to investigate the relative expression of N transport-related genes in the root of *Eucalyptus* (Zhao et al., 2019). The samples were taken in liquid nitrogen and transferred to -80°C refrigerator for storage. RNA was extracted using E.Z.N.A.™ Plant RNA Protocol II kit (Guangzhou Omega Biotek, Ltd). HiScript^®^ III RT SuperMix for qPCR (+gDNA wiper) kit (Nanjing Vazyme Biotech Co., Ltd) was used to reverse transcription of the first strand cDNA. Furthermore, gene expression levels were determined in a 96-well Real time PCR system instrument (BioRed, Hercules, CA, USA), using the SGExcel FastSYBR Master Mix kit (Shanghai Sangon Biotech Co., Ltd.), with a reaction mixture of 10 µL SYBR qPCR Master Mix, 0.5 µL each of forward and reverse primers, 2 µL cDNA, and 7 µL ddH_2_O. Nine primers were selected to be used in the qRT-PCR, with *EgUBI3* applied to be the endogenous reference (Huang et al., 2014). Each PCR protocol was conducted with 3 biological replicates. **Supplementary Table 1** presents all the primers used in the qRT-PCR.

### 2.5 Statistical analysis

In the current work, SPSS 27.0 (SPSS Inc., Chicago, IL, USA) was adopted for statistical analysis of the result data. The result data were analyzed based on one-way ANOVA (posthoc comparisons using Duncan’s test, *P* < 0.05) and two-way ANOVA (the variation sources were AM fungi inoculation, N application, P application, organic N and their association). T-test was analyzed between NM and AM treatments. After performing the homogeneity analysis of variance, the data were subjected to ANOVA. Pearson’s correlation coefficient was utilized to evaluate the correlation between different growth and physiological characters. Origin 8.0 software (Systat Software Inc., San Jose, CA, USA) was employed to construct figures.

## 3 Results

### 3.1 Growth of mycorrhizal *Eucalyptus* seedlings under different organic N and P conditions

**Figure 1:**
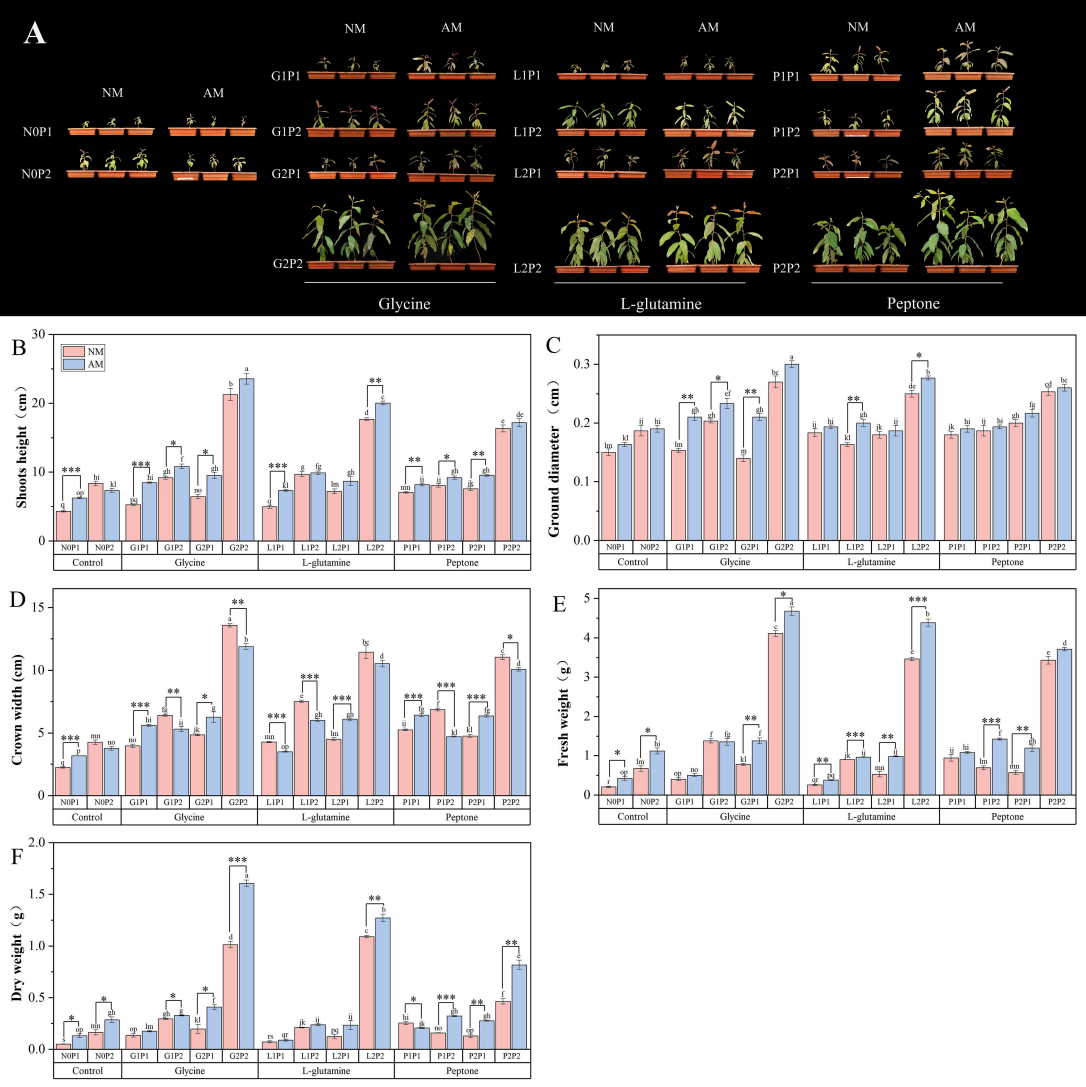
Mycorrhizal *Eucalyptus* seedlings growth under different organic N and P treatments. Growth phenotypes **(A)**, height **(B)**, ground diameter **(C)**, crown width **(D)**, dry weight **(E)**, fresh weight **(F)**. N0P1: 1 mM N-NO_3_^-^ and 0.03 mM Pi treatment; N0P2: 1 mM N-NO_3_^-^ and 0.3 mM Pi treatment; G1P1: 0.25 mM N-NO_3_^-^, 0.25 mM glycine and 0.03 mM Pi treatment; G1P2: 0.25 mM N-NO_3_^-^, 0.25 mM glycine and 0.3 mM Pi treatment; G2P1: 0.25 mM N-NO_3_^-^, 2.5 mM glycine and 0.03 mM Pi treatment; G2P2: 0.25 mM N-NO_3_^-^, 2.5 mM glycine and 0.3 mM Pi treatment; L1P1: 0.25 mM N-NO_3_^-^, 0.25 mM L-glutamine and 0.03 mM Pi treatment; L1P2: 0.25 mM N-NO_3_^-^, 0.25 mM L-glutamine and 0.3 mM Pi treatment; L2P1: 0.25 mM N-NO_3_^-^, 2.5 mM L-glutamine and 0.03 mM Pi treatment; L2P2: 0.25 mM N-NO_3_^-^, 2.5 mM L-glutamine and 0.3 mM Pi treatment; P1P1: 0.25 mM N-NO_3_^-^, 0.25 mM peptone and 0.03 mM Pi treatment; P1P2: 0.25 mM N-NO_3_^-^, 0.25 mM peptone and 0.3 mM Pi treatment; P2P1: 0.25 mM N-NO_3_^-^, 2.5 mM peptone and 0.03 mM Pi treatment; P2P2: 0.25 mM N-NO_3_^-^, 2.5 mM peptone and 0.3 mM Pi treatment. NM, non-mycorrhizal treatment; AM, mycorrhizal treatment. Control, no organic N treatment. Glycine, glycine treatment; L-glutamine, L-glutamine treatment; Peptone, peptone treatment. Using Duncan test for inter group differences, different letters meant that the factors changed significantly among the four strategies (*P* < 0.05). Using t-test between NM and AM treatments, *P < 0.05 level is significant; **P < 0.01 level is very significant; ***P < 0.001 level is extremely significant.

The G2P2 treatment showed the most notable increase in *Eucalyptus* growth among various treatments. *Eucalyptus* seedlings treated with G2P2 had heights, ground diameters, and crown widths that were 3.23, 0.82, and 3.70 times higher than N0P1 treatment, respectively. Compared to non-mycorrhizal *Eucalyptus* seedlings, mycorrhizal *Eucalyptus* seedlings under both N1P1 and N2P1 treatments showed a substantial increase in plant height and crown width with glycine and peptone treatments (**Figures 1B, D**). The results indicated that glycine outperformed NM treatments among the three organic N treatments in terms of promoting mycorrhizal *Eucalyptus* growth (**Figure 1**). Specifically, the height and crown width of mycorrhizal *Eucalyptus* seedlings treated with G1P1 were elevated by 60.72% and 41.56%, compared to NM treatments (**Figures 1B, C**). This implies that at the same N level, mycorrhizal *Eucalyptus* has a considerable growth advantage in low P conditions. However, the growth of *Eucalyptus* seedlings did not significantly increase after high N treatments at the same P level, suggesting that P is a more important factor restricting *Eucalyptus* seedling growth (**Figure 1**).

The dry weight and fresh weight of mycorrhizal *Eucalyptus* seedlings treated with high organic N were found to be considerably higher than NM treatments. In particular, the dry weight of mycorrhizal *Eucalyptus* seedlings treated with G2P2 was increased by 58.79% compared to NM treatment (**Figure 1F**). Among the three organic N sources, glycine treatment led to a considerable increase in seedling biomass. There were substantial increases in seedling biomass under N2P2 treatments, the dry weights and fresh weights of *Eucalyptus* under G2P2 treatment were 13.39 and 12.93 times, respectively, higher than those under N0P1 treatment (**Figures 1E, F**). This suggests that *Eucalyptus* seedling biomass was more effectively promoted by higher levels of N and P. In terms of increasing the biomass of *Eucalyptus* seedlings, the G2P2 treatment performed the best overall.

### 3.2 Root growth of mycorrhizal *Eucalyptus* seedlings under different organic N and P conditions

**Figure 2:**
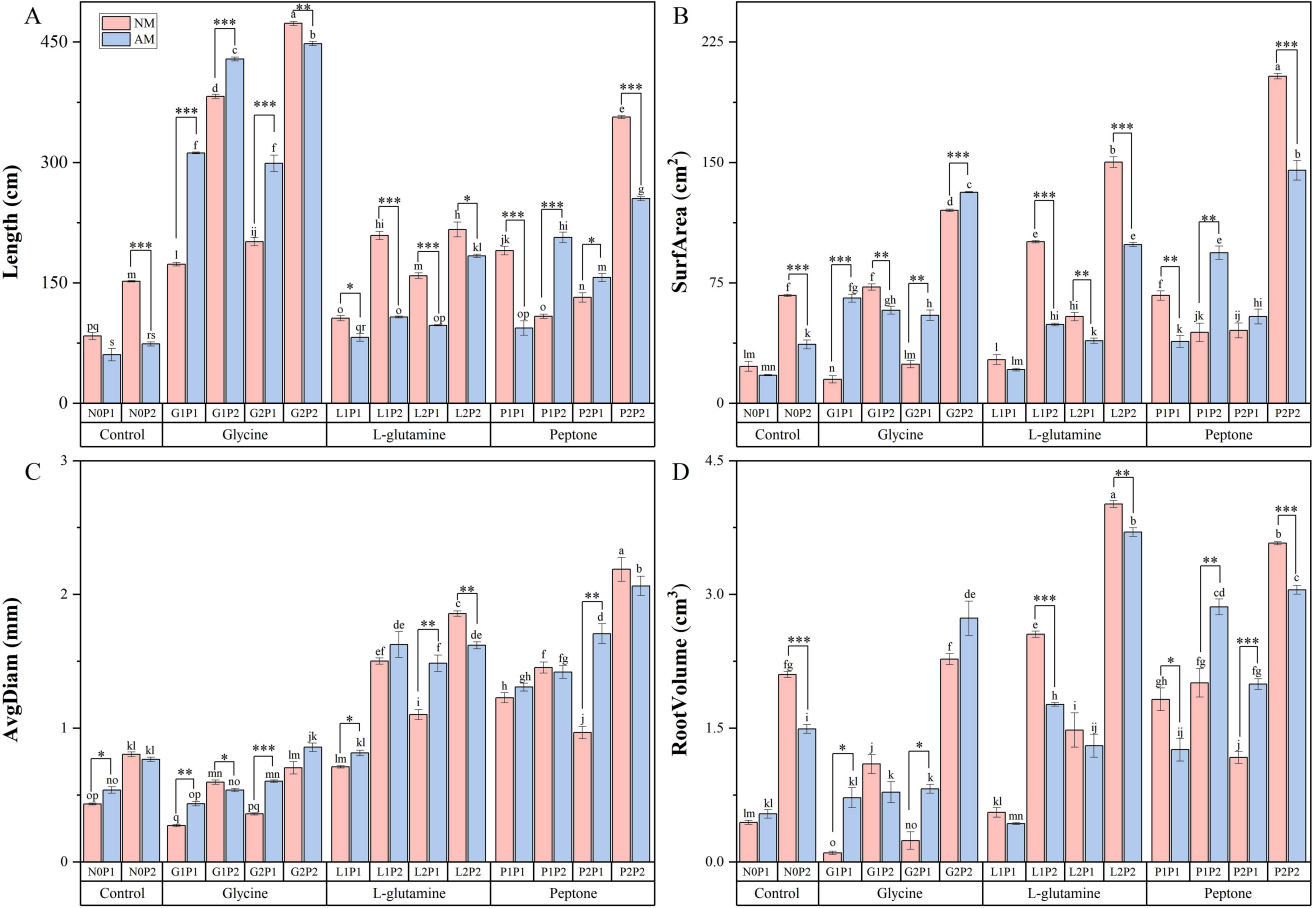
Mycorrhizal *Eucalyptus* seedlings root growth under different organic N and P treatments. Roots length **(A)**, surfarea **(B)**, avgdiam **(C)**, root volume **(D)**. N0P1: 1 mM N-NO_3_^-^ and 0.03 mM Pi treatment; N0P2: 1 mM N-NO_3_^-^ and 0.3 mM Pi treatment; G1P1: 0.25 mM N-NO_3_^-^, 0.25 mM glycine and 0.03 mM Pi treatment; G1P2: 0.25 mM N-NO_3_^-^, 0.25 mM glycine and 0.3 mM Pi treatment; G2P1: 0.25 mM N-NO_3_^-^, 2.5 mM glycine and 0.03 mM Pi treatment; G2P2: 0.25 mM N-NO_3_^-^, 2.5 mM glycine and 0.3 mM Pi treatment; L1P1: 0.25 mM N-NO_3_^-^, 0.25 mM L-glutamine and 0.03 mM Pi treatment; L1P2: 0.25 mM N-NO_3_^-^, 0.25 mM L-glutamine and 0.3 mM Pi treatment; L2P1: 0.25 mM N-NO_3_^-^, 2.5 mM L-glutamine and 0.03 mM Pi treatment; L2P2: 0.25 mM N-NO_3_^-^, 2.5 mM L-glutamine and 0.3 mM Pi treatment; P1P1: 0.25 mM N-NO_3_^-^, 0.25 mM peptone and 0.03 mM Pi treatment; P1P2: 0.25 mM N-NO_3_^-^, 0.25 mM peptone and 0.3 mM Pi treatment; P2P1: 0.25 mM N-NO_3_^-^, 2.5 mM peptone and 0.03 mM Pi treatment; P2P2: 0.25 mM N-NO_3_^-^, 2.5 mM peptone and 0.3 mM Pi treatment. NM, non-mycorrhizal treatment; AM, mycorrhizal treatment. Control, no organic N treatment. Glycine, glycine treatment; L-glutamine, L-glutamine treatment; Peptone, peptone treatment. Using Duncan test for inter group differences, different letters meant that the factors changed significantly among the four strategies (*P* < 0.05). Using t-test between NM and AM treatments, *P < 0.05 level is significant; **P < 0.01 level is very significant; ***P < 0.001 level is extremely significant.

Analysis of *Eucalyptus* seedlings root growth revealed significant increases in root length under glycine treatments (**Figure 2A**). Root length under G2P2 treatment was 5.38 times higher than that of N0P1 treatment. The root surface area was increased by 7.60 times under P2P2 treatment compared to N0P1 treatment (**Figure 2B**). Concurrently, the root average diameter and volume were significantly increased under L-glutamine treatments, compared to control treatment (**Figures 2C, D**). The average root volume of mycorrhizal *Eucalyptus* was elevated by 6.07 times compared to non-mycorrhizal *Eucalyptus* under G1P1 treatment. However, non-mycorrhizal *Eucalyptus* seedlings exhibited superior root growth, compared to mycorrhizal *Eucalyptus* seedlings under N2P2 treatment. Furthermore, compared to NM treatments, glycine treatment was more successful in lengthening the mycorrhizal *Eucalyptus* roots. Under high P treatments, root growth significantly increased in comparison to treatments at the same N level (**Figure 2**). The root length of G2P2 treatment was increased by 0.84 times compared to G2P1 treatment, indicating that P plays a significant role in the root growth of *Eucalyptus* seedlings. There was an overall increase in root growth under P2P2 treatment. Compared to N0P1 treatment, the root length, surface area, average diameter and volume of *Eucalyptus* seedlings were increased by 3.23, 7.60, 3.38 and 5.76 times, accordingly. Overall, P2P2 treatment was the best in enhancing the *Eucalyptus* root growth.

### 3.3 Photosynthetic characteristic of mycorrhizal *Eucalyptus* seedlings under different organic N and P conditions

**Figure 3:**
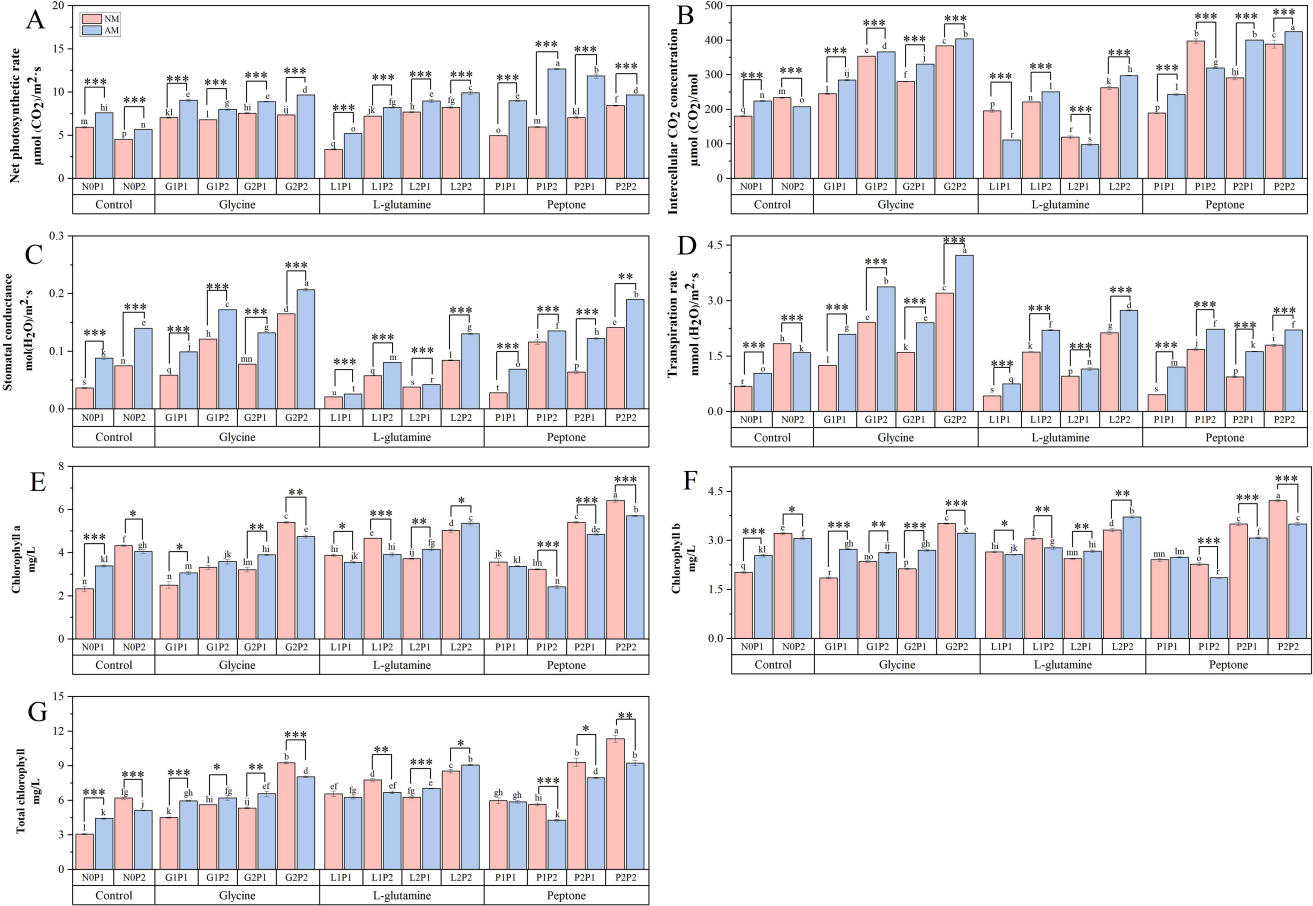
Mycorrhizal *Eucalyptus* seedlings photosynthetic characteristic under different organic N and P treatments. Net photosynthetic rate **(A)**, intercellular CO_2_ concentration **(B)**, stomatal conductance **(C)**, transpiration rate **(D)**, chlorophyll a concentration **(E)**, chlorophyll b concentration **(F)**, total chlorophyll concentration **(G)**. N0P1: 1 mM N-NO_3_^-^ and 0.03 mM Pi treatment; N0P2: 1 mM N-NO_3_^-^ and 0.3 mM Pi treatment; G1P1: 0.25 mM N-NO_3_^-^, 0.25 mM glycine and 0.03 mM Pi treatment; G1P2: 0.25 mM N-NO_3_^-^, 0.25 mM glycine and 0.3 mM Pi treatment; G2P1: 0.25 mM N-NO_3_^-^, 2.5 mM glycine and 0.03 mM Pi treatment; G2P2: 0.25 mM N-NO_3_^-^, 2.5 mM glycine and 0.3 mM Pi treatment; L1P1: 0.25 mM N-NO_3_^-^, 0.25 mM L-glutamine and 0.03 mM Pi treatment; L1P2: 0.25 mM N-NO_3_^-^, 0.25 mM L-glutamine and 0.3 mM Pi treatment; L2P1: 0.25 mM N-NO_3_^-^, 2.5 mM L-glutamine and 0.03 mM Pi treatment; L2P2: 0.25 mM N-NO_3_^-^, 2.5 mM L-glutamine and 0.3 mM Pi treatment; P1P1: 0.25 mM N-NO_3_^-^, 0.25 mM peptone and 0.03 mM Pi treatment; P1P2: 0.25 mM N-NO_3_^-^, 0.25 mM peptone and 0.3 mM Pi treatment; P2P1: 0.25 mM N-NO_3_^-^, 2.5 mM peptone and 0.03 mM Pi treatment; P2P2: 0.25 mM N-NO_3_^-^, 2.5 mM peptone and 0.3 mM Pi treatment. NM, non-mycorrhizal treatment; AM, mycorrhizal treatment. Control, no organic N treatment. Glycine, glycine treatment; L-glutamine, L-glutamine treatment; Peptone, peptone treatment. Using Duncan test for inter group differences, different letters meant that the factors changed significantly among the four strategies (*P* < 0.05). Using t-test between NM and AM treatments, *P < 0.05 level is significant; **P < 0.01 level is very significant; ***P < 0.001 level is extremely significant.

There was no discernible increase in the net photosynthetic rate with the three organic N treatments. However, the net photosynthetic rate was increased by 0.38 and 0.40 times, respectively, under P1P2 and P2P1 treatment compared to N0P1 treatment (**Figure 3A**). Under the P1P2 treatment, net photosynthetic rate of mycorrhizal *Eucalyptus* seedlings was elevated by 113.10%, compared to NM treatment. Both the glycine and peptone treatments resulted in a considerable rise in the intercellular CO_2_ concentration (**Figure 3B**). Under glycine treatment, stomatal conductance and transpiration rate increased significantly. Compared to N0P1 treatment, the stomatal conductance and transpiration rate under G2P2 treatment were increased by 1.92 and 3.33 times, respectively (**Figures 3C, D**). Furthermore, *Eucalyptus* net photosynthesis rate, intercellular CO_2_ concentration, stomatal conductance, and transpiration rate were all increased under high P treatments in comparison to treatments at the same N level, suggesting that P is also a crucial component in boosting *Eucalyptus* seedling photosynthesis. The photosynthesis of *Eucalyptus* seedlings under the G2P2 treatment were increased notably. Overall, these results showed that glycine was superior in encouraging photosynthesis among the three organic N treatments.

Chlorophyll concentrations in *Eucalyptus* seedlings were shown to increase following P2P2 treatment over various treatments (**Figures 3E–G**). The concentrations of chlorophyll a, chlorophyll b and total chlorophyll under P2P2 treatment were 1.12, 0.69 and 1.75 times, accordingly, higher than those of N0P1 treatment. Also, the chlorophyll concentrations under treatments with high N and P increased significantly, revealing that high level of N and P increased the *Eucalyptus* seedlings’ chlorophyll concentrations. The findings demonstrated that mycorrhizal *Eucalyptus* seedlings treated with glycine had a higher chlorophyll concentration than non-mycorrhizal *Eucalyptus* seedlings. However, these results contrasted under G2P2 treatment, implying that higher level of N and P restricted the increase of mycorrhizal *Eucalyptus* seedlings’ chlorophyll concentrations.

### 3.4 Enzyme activities related to N metabolism and total N P content under different organic N and P conditions

**Figure 4:**
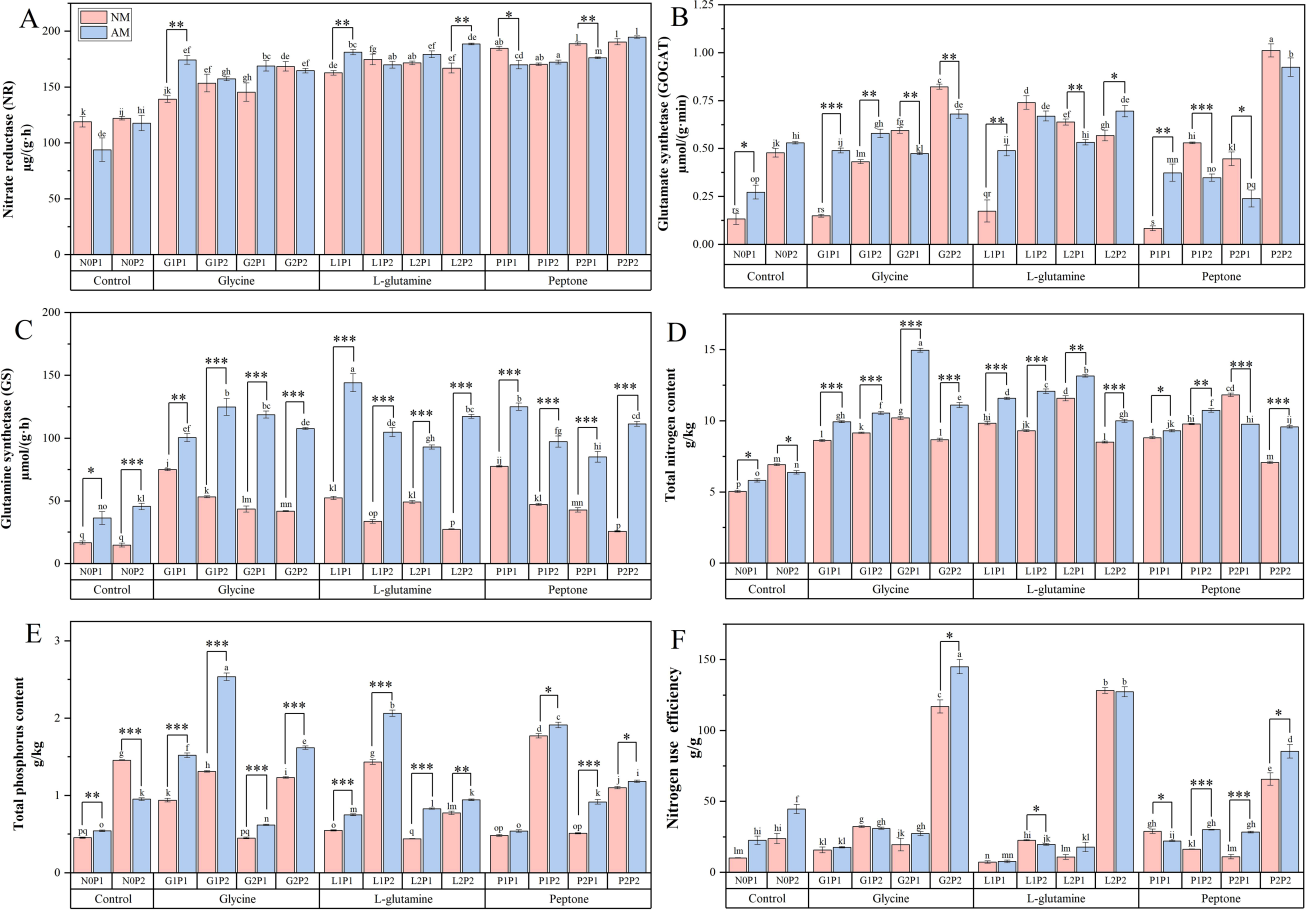
Mycorrhizal *Eucalyptus* seedlings N metabolism-related enzymes activities, TN TP contents and nitrogen use efficiency under different organic N and P treatments. Activity of nitrate reductase **(A)**, activity of glutamate synthetase **(B)**, activity of glutamine synthetase **(C)**, total nitrogen content **(D)**, total phosphorus content **(E)**, nitrogen use efficiency **(F)**. N0P1: 1 mM N-NO_3_^-^ and 0.03 mM Pi treatment; N0P2: 1 mM N-NO_3_^-^ and 0.3 mM Pi treatment; G1P1: 0.25 mM N-NO_3_^-^, 0.25 mM glycine and 0.03 mM Pi treatment; G1P2: 0.25 mM N-NO_3_^-^, 0.25 mM glycine and 0.3 mM Pi treatment; G2P1: 0.25 mM N-NO_3_^-^, 2.5 mM glycine and 0.03 mM Pi treatment; G2P2: 0.25 mM N-NO_3_^-^, 2.5 mM glycine and 0.3 mM Pi treatment; L1P1: 0.25 mM N-NO_3_^-^, 0.25 mM L-glutamine and 0.03 mM Pi treatment; L1P2: 0.25 mM N-NO_3_^-^, 0.25 mM L-glutamine and 0.3 mM Pi treatment; L2P1: 0.25 mM N-NO_3_^-^, 2.5 mM L-glutamine and 0.03 mM Pi treatment; L2P2: 0.25 mM N-NO_3_^-^, 2.5 mM L-glutamine and 0.3 mM Pi treatment; P1P1: 0.25 mM N-NO_3_^-^, 0.25 mM peptone and 0.03 mM Pi treatment; P1P2: 0.25 mM N-NO_3_^-^, 0.25 mM peptone and 0.3 mM Pi treatment; P2P1: 0.25 mM N-NO_3_^-^, 2.5 mM peptone and 0.03 mM Pi treatment; P2P2: 0.25 mM N-NO_3_^-^, 2.5 mM peptone and 0.3 mM Pi treatment. NM, non-mycorrhizal treatment; AM, mycorrhizal treatment. Control, no organic N treatment. Glycine, glycine treatment; L-glutamine, L-glutamine treatment; Peptone, peptone treatment. Using Duncan test for inter group differences, different letters meant that the factors changed significantly among the four strategies (*P* < 0.05). Using t-test between NM and AM treatments, *P < 0.05 level is significant; **P < 0.01 level is very significant; ***P < 0.001 level is extremely significant.

Under different treatments, the activities of NR and GOGAT were significantly elevated under the P2P2 treatment. Compared to N0P1 treatment, NR and GOGAT activities under P2P2 treatment in *Eucalyptus* seedlings were increased by 0.81 and 3.82 times, respectively (**Figures 4A, B**). GS activity in mycorrhizal *Eucalyptus* was increased significantly, particularly under L1P1 treatment, where it was 1.75 times higher than NM treatment (**Figure 4C**). The activities of NR, GOGAT and GS in mycorrhizal *Eucalyptus* seedlings were elevated under G1P1 and G1P2 treatments compared to NM treatment (**Figure 4**). High P treatments did not effectively promote the GS activity in *Eucalyptus*. Concurrently, activity of NR and GOGAT under G1P1 treatment was 0.25 and 2.27 times, respectively, higher than NM treatments, indicating that high N and P levels did not promote the activities of NR and GOGAT. The results demonstrated that among the three organic N treatments, glycine treatment was more effective in promoting N metabolism in mycorrhizal *Eucalyptus* compared to non-mycorrhizal *Eucalyptus* (**Figure 4**).

The TN content in *Eucalyptus* seedlings was significantly elevated under G2P1 treatment. The TN content in mycorrhizal *Eucalyptus* seedlings under N2P1 treatment was increased by 0.47 times compared to NM treatment (**Figure 4D**). Additionally, compared to N0P1 treatment, the TN content in *Eucalyptus* was 0.67 times higher than that of G2P1 treatment. While high P treatments were less effective, high N fertilization significantly raised the TN contents. Concurrently, the TP content in mycorrhizal *Eucalyptus* seedlings treated with G1P2 was elevated by 93.13%, compared to non-mycorrhizal *Eucalyptus* (**Figure 4E**). Additionally, high N level had no discernible effect on the TP contents. Under various treatments, the N2P2 treatments was the most effective in promoting the NUE of mycorrhizal *Eucalyptus*. The mycorrhizal *Eucalyptus* showed notable benefits in boosting NUE when exposed to high levels of organic N fertilization, particularly glycine treatments. NUE of mycorrhizal *Eucalyptus* under G2P2 treatment was elevated by 24.03%, compared to non-mycorrhizal *Eucalyptus*. The results indicated that among the three organic N treatments, glycine treatment was more effective in increasing the TN contents, TP contents and NUE of mycorrhizal *Eucalyptus* compared to non-mycorrhizal *Eucalyptus* (**Figure 4**).

### 3.5 Relative expression of genes related N transport of mycorrhizal *Eucalyptus* seedlings under different organic N and P conditions

**Figure 5:**
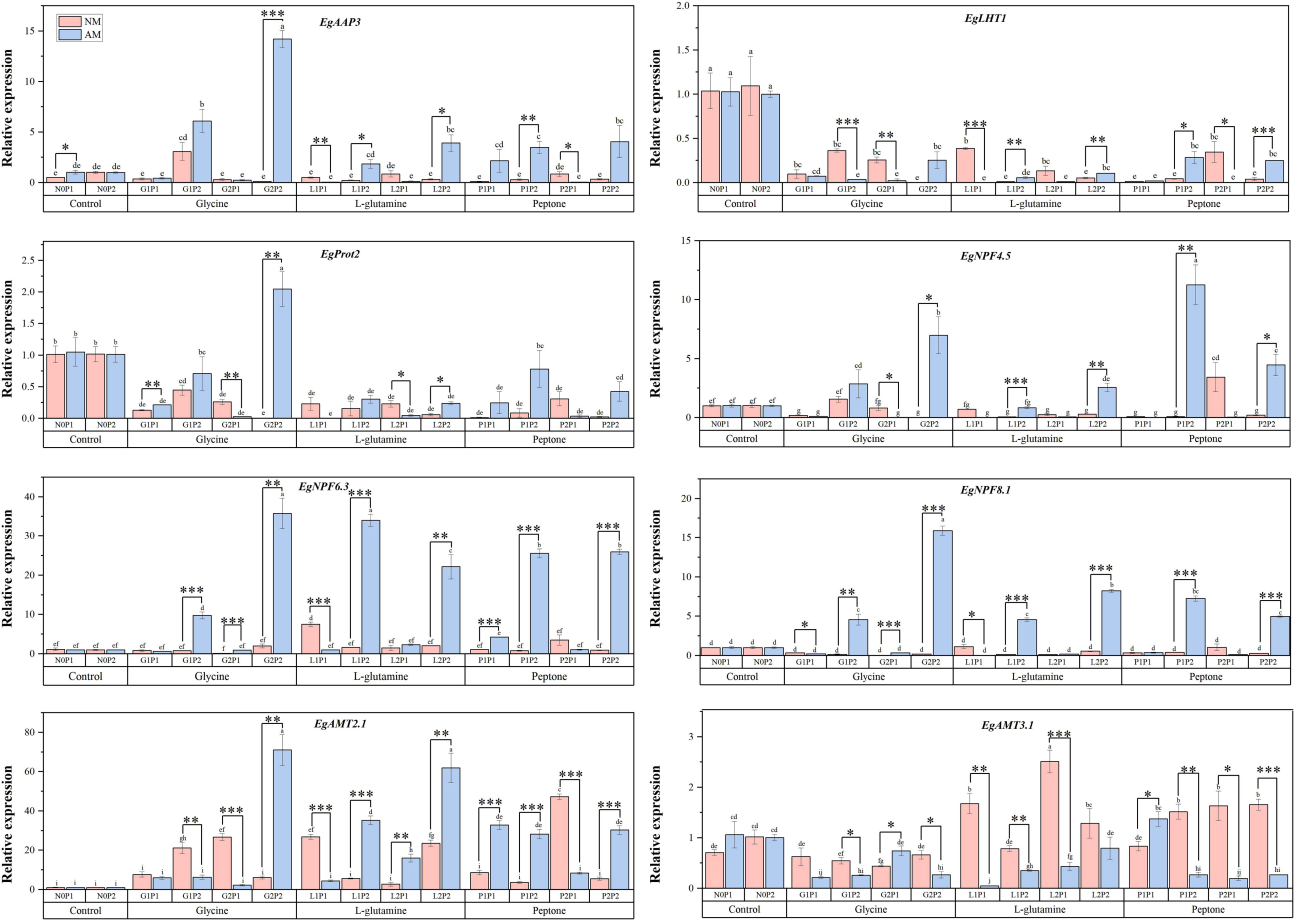
Mycorrhizal *Eucalyptus* seedlings relative expressions of genes related to N transport under different organic N and P treatments. N0P1: 1 mM N-NO_3_^-^ and 0.03 mM Pi treatment; N0P2: 1 mM N-NO_3_^-^ and 0.3 mM Pi treatment; G1P1: 0.25 mM N-NO_3_^-^, 0.25 mM glycine and 0.03 mM Pi treatment; G1P2: 0.25 mM N-NO_3_^-^, 0.25 mM glycine and 0.3 mM Pi treatment; G2P1: 0.25 mM N-NO_3_^-^, 2.5 mM glycine and 0.03 mM Pi treatment; G2P2: 0.25 mM N-NO_3_^-^, 2.5 mM glycine and 0.3 mM Pi treatment; L1P1: 0.25 mM N-NO_3_^-^, 0.25 mM L-glutamine and 0.03 mM Pi treatment; L1P2: 0.25 mM N-NO_3_^-^, 0.25 mM L-glutamine and 0.3 mM Pi treatment; L2P1: 0.25 mM N-NO_3_^-^, 2.5 mM L-glutamine and 0.03 mM Pi treatment; L2P2: 0.25 mM N-NO_3_^-^, 2.5 mM L-glutamine and 0.3 mM Pi treatment; P1P1: 0.25 mM N-NO_3_^-^, 0.25 mM peptone and 0.03 mM Pi treatment; P1P2: 0.25 mM N-NO_3_^-^, 0.25 mM peptone and 0.3 mM Pi treatment; P2P1: 0.25 mM N-NO_3_^-^, 2.5 mM peptone and 0.03 mM Pi treatment; P2P2: 0.25 mM N-NO_3_^-^, 2.5 mM peptone and 0.3 mM Pi treatment. NM, non-mycorrhizal treatment; AM, mycorrhizal treatment. Control, no organic N treatment. Glycine, glycine treatment; L-glutamine, L-glutamine treatment; Peptone, peptone treatment. Using Duncan test for inter group differences, different letters meant that the factors changed significantly among the four strategies (*P* < 0.05). Using t-test between NM and AM treatments, *P < 0.05 level is significant; **P < 0.01 level is very significant; ***P < 0.001 level is extremely significant.

Under different treatments, the relative expression levels of genes associated with N transport in mycorrhizal *Eucalyptus* significantly increased under G2P2 treatment (**Figure 5**). The AM treatment markedly elevated the relative expression levels of *EgAAP3*, *EgProt2*, *EgNPF4.5*, *EgNPF6.3*, *EgNPF8.1* and *EgAMT2.1*, compared to NM treatments under three organic N treatments. The relative expression of genes related to organic N transport (*EgAAP3* and *EgProt2*), nitrate N transport (*EgNPF4.5*, *EgNPF6.3* and *EgNPF8.1*) in mycorrhizal *Eucalyptus* were significantly up-regulated under high P treatments compared to treatments at the same organic N level. This suggests that P may be a key factor regulating nitrate N transport and amino acid transport in *Eucalyptus* (**Figure 5**). Under G2P2 and L2P2 treatments, mycorrhizal *Eucalyptus* showed considerably greater relative expression levels of *EgAMT2.1* than under NM treatments, whereas under L1P1 and L2P1 treatments, mycorrhizal *Eucalyptus* showed significantly lower relative expression levels of *EgAMT3.1* than under NM treatments. This might indicate that plants use distinct ammonium ion transporter proteins to control ammonium ion absorption under various N supply situations.

### 3.6 Correlation analysis between different growth and physiological characters of *Eucalyptus* seedlings

**Figure 6:**
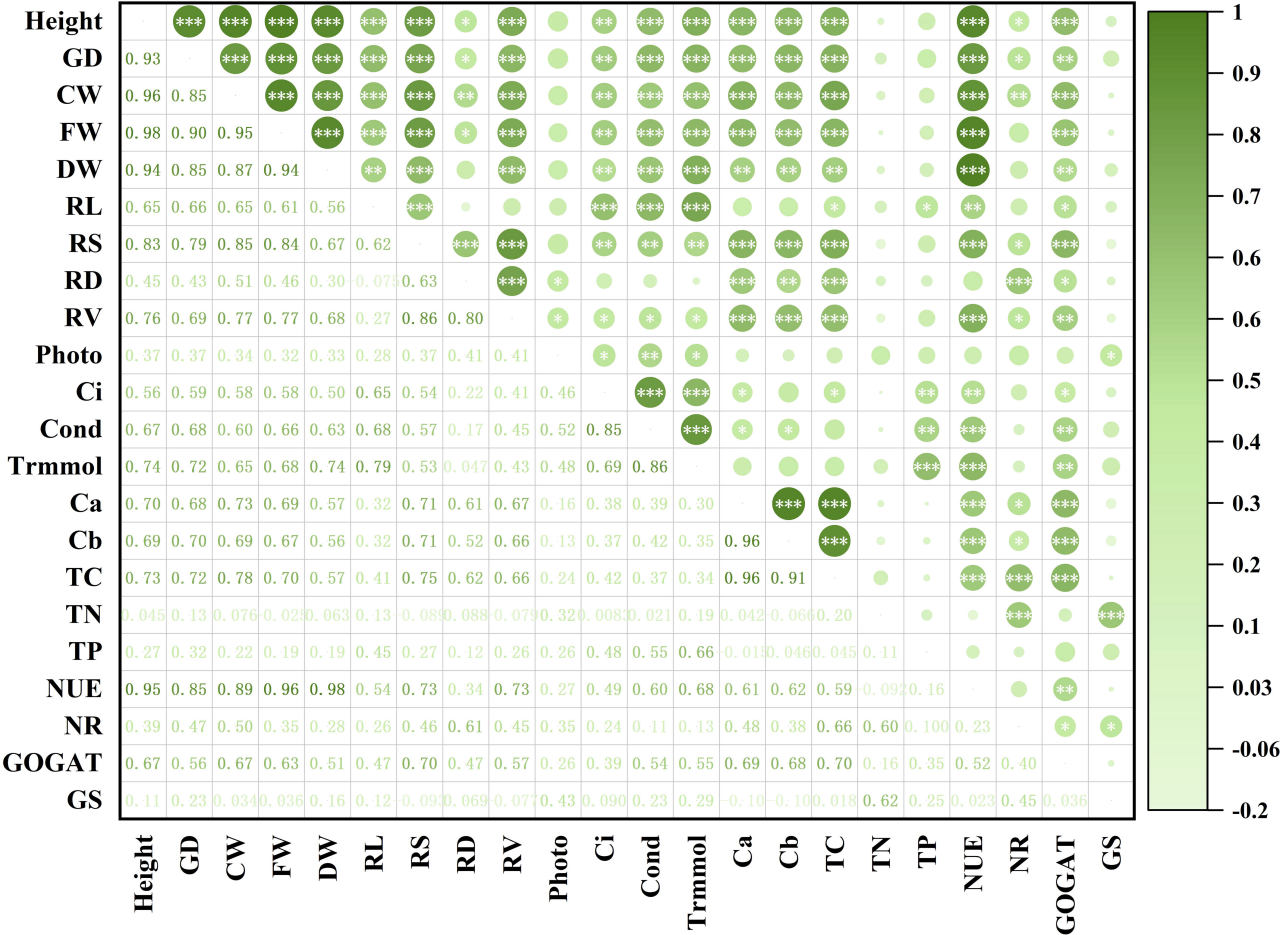
Correlation analysis between different growth and physiological characters of *Eucalyptus*. Height: seedling height; GD: ground diameter; CW: crown width; FW: fresh weight; DW: dry weight; RL: root length; RS: root average surface area; RD: root average diameter; RV: root average volume; Photo: net photosynthetic rate; Ci: intercellular CO_2_ concentration; Cond: stomatal conductance; Trmmol: transpiration rate; C_a_: chlorophyll a concentration; C_b_: chlorophyll b concentration; TC: total chlorophyll concentration; TN: total nitrogen content; TP: total phosphorus content; NUE: nitrogen use efficiency; NR: activity of nitrate reductase; GOGAT: activity of glutamate synthetase; GS: activity of glutamine synthetase. The dark green color represents a positive correlation between the two indicators, and light green represents a negative correlation between the two indicators, and the depth of the color represents the level of correlation. An asterisk (*) represents that the correlation between the two indicators reaches a significant difference level *(P* < 0.05), two asterisks (**) represent that the correlation between the two indicators reaches a very significant difference level (*P* < 0.01), three asterisks (***) represent that the correlation between the two indicators reaches an extremely significant difference level (*P* < 0.001).

Significant positive relationships between seedling growth features and photosynthetic characteristics were found based on the correlation plot among several *Eucalyptus* growth and physiological characters (**Figure 6**). This suggests that photosynthesis is a major factor influencing seedling growth. Chlorophyll concentration and seedling growth characteristics showed highly significant positive associations with NUE (*P* < 0.001). Additionally, TC was shown to have highly significant positive associations (*P* < 0.001) with both NR and GOGAT activities. In summary, GOGAT activity, photosynthesis, and NUE are important variables affecting *Eucalyptus* growth. As presented in **Supplementary Table 2**, the impacts of AM fungi, N application and P application, organic N and the interactions of the previous four significantly (*P* < 0.01) elevated the levels of evaluated growth and photosynthesis indexes.

## 4 Discussion

### 4.1 Effects of AM fungi on plant growth under different N and P conditions

AM fungi play a vital role in enhancing plant growth under nutrient-limited conditions. Our study revealed that AM treatments exhibited significantly greater biomass accumulation, compared to NM treatments, with low P fertilization under the same N level (**Figure 1**). This aligns with previous observations that AM fungi symbiosis improves P acquisition efficiency in P deficient soil by extending the root absorption zone, thereby compensating for reduced root hair development (Qian et al., 2024). Notably, this growth advantage diminished under N2P2 treatments, where NM treatments outperformed their AM treatments, likely due to suppressed AM fungi colonization when P availability exceeds plant requirements (**Figure 2**).

The AM fungi symbiosis further modulated seedlings’ organic N preference by enhancing NUE. Among three organic N sources, glycine emerged as the most effective in promoting mycorrhizal seedlings growth, contrasting with limited benefits observed in NM treatments. This finding corroborates evidence that forest-dominant plants, regardless of mycorrhizal type, preferentially acquire low-molecular-weight organic N compounds like glycine (Näsholm et al., 1998; Naz et al., 2024). Glycine’s efficiency in promoting *Eucalyptus* growth may be due to its small molecular weight and simple structure, making it easily absorbed by plants directly. The molecular simplicity of glycine likely facilitates direct uptake through AM-mediated pathways, bypassing energy-intensive mineralization processes required for complex N forms.

A synergistic mechanism between AM fungi and organic N was evident through coordinated nutrient interactions. While N fertilization independently promoted root proliferation, AM fungi inoculation amplified this effect by improving P co-utilization efficiency (Chen et al., 2020). The symbiosis induced physiological modifications in root architecture and exudation patterns, creating feedback loops that enhanced both N and P acquisition (Mazumder et al., 2025). This synergy was particularly pronounced under moderate fertilization regimes, where AM fungi optimized the balance between N assimilation and P mobilization. Our results demonstrate that AM-mediated nutrient coordination, rather than isolated nutrient applications, drives sustainable *Eucalyptus* growth, which is a critical insight for developing precision fertilization strategies in forest management.

### 4.2 Effects of AM fungi on plant photosynthetic characteristic and total N P contents under different N and P conditions

In our study, treatments with high N and P significantly increased the seedlings chlorophyll concentration (**Figure 3**), which corresponds with the previous finding that leaf chlorophyll content is positively correlated with N supply level. Increasing N supply typically elevates leaf chlorophyll content (Wu et al., 2017b; Chen et al., 2024). At the same time, AM fungi significantly improved photosynthetic efficiency of *Eucalyptus* seedlings under P-limited conditions. While high P treatments universally boosted net photosynthesis, stomatal conductance, and transpiration rate (**Figure 3**), AM treatments exhibited superior photosynthetic performance compared to NM treatments under low P availability. This aligns with P’s critical role as a substrate, and deficiency or low level of P inhibit leaf photosynthetic rates (Yu et al., 2022). Notably, AM fungi symbiosis optimized N partitioning under low N conditions, redirecting N resources from structural components to photosynthetic machinery, thereby amplifying light energy capture and carbon assimilation (Wu et al., 2017a). This reallocation mechanism underscores AM fungi’s ability to mitigate nutrient limitations by prioritizing metabolic processes essential for growth under low fertilization (Sun J, et al., 2024).

The symbiosis between AM fungi and *Eucalyptus* selectively enhanced organic N utilization, with glycine emerging as the most effective N source for mycorrhizal seedlings. Under glycine treatments, AM treatments showed markedly elevated chlorophyll concentrations compared to NM treatments (**Figure 3**), a response linked to enhanced NUE and upregulated activities of N metabolism enzymes (NR, GOGAT) (**Figure 6**). These findings corroborate that AM fungi facilitate direct assimilation of low-molecular-weight organic N compounds, bypassing energy-intensive mineralization pathways (Näsholm et al., 1998; Naz et al., 2024). By increasing chlorophyll biosynthesis, AM fungi amplified capacity on harvesting light, creating a positive feedback loop where improved N metabolism reinforced photosynthetic output. This preferential utilization of glycine highlights AM fungi’s role in fine-tuning N source selection to maximize host plant productivity.

Several studies have demonstrated that AM fungi can facilitate plant uptake of soil nutrients such as N and P, increasing plant biomass, N concentration, P concentration, N uptake of whole plant (Che et al., 2022; Wu Y, et al., 2024). Our findings observed that high P treatments behaved better in increasing mycorrhizal *Eucalyptus*’s TP contents. This effect is most likely mediated by improved P acquisition in mycorrhizal plants compared to non-mycorrhizal plants under low P conditions. Outsourcing P acquisition to mycorrhiza may limit the value of P loss reduction by plants (Wang D, et al., 2025). AM fungi-organic N synergy drove coordinated improvements in N and P homeostasis. AM treatments exhibited simultaneous increases in TN and TP contents (**Figure 4**), reflecting synergistic interactions exist between N and P uptake. While AM fungi primarily enhanced P uptake via extraradical hyphae that bypass rhizosphere depletion zones (Smith et al., 2011; Bhupenchandra et al., 2024), organic N fertilization indirectly boosted P availability by stimulating soil phosphatase activity, accelerating organic P mineralization (Gou et al., 2024). This dual mechanism direct AM-mediated P scavenging and N-driven P mobilization, creating a synergistic nutrient loop. High P treatments amplified this interaction, as AM fungi colonization under sufficient P conditions optimized N-P stoichiometry, further elevating chlorophyll synthesis and photosynthetic rates. Such interdependence underscores that AM fungi do not merely supplement nutrient uptake but actively rewire host plant metabolism to exploit nutrient co-limitation, transforming discrete nutrient inputs into multiplicative growth benefits.

### 4.3 Effects of AM fungi on plant activity of enzymes related to N metabolism under different N and P conditions

AM fungi significantly upregulated key N metabolic enzymes in *Eucalyptus* leaves, even under N-deficient conditions. Mycorrhizal seedlings exhibited elevated activities of NR, GOGAT, and GS, compared to NM treatments (**Figure 4**), consistent with AM fungi’s role in enhancing N uptake and assimilation. The GS-GOGAT cycle, which is critical for converting ammonium into glutamine and glutamate, was particularly amplified, thus providing precursors for amino acids, nucleic acids, and chlorophyll synthesis. This aligns with studies showing AM fungi colonization upregulates genes encoding nitrate transporters and N metabolism enzymes, enabling efficient N recycling under low nutrient conditions (Wang et al., 2020; Chen et al., 2023; Wu XL, et al., 2024). Notably, this enzymatic enhancement occurred independently of fertilization levels, suggesting AM fungi constitutively prime N assimilation pathways to mitigate nutrient scarcity.

The symbiosis selectively optimized organic N utilization by prioritizing ammonium assimilation via the GS-GOGAT pathway (Fortunato et al., 2023; Vaishnav et al., 2025). While NM treatments relied heavily on soil nitrate, mycorrhizal *Eucalyptus* exhibited a metabolic shift toward glutamine synthesis, a hallmark of organic N preference. This reprogramming explains why mycorrhizal seedlings maintained higher GS activity across all treatments (**Figure 4**), as GS catalyzes the ATP-dependent fixation of ammonium, which is the key step in assimilating organic N breakdown products (Kojima et al., 2021). By bypassing energetically costly nitrate reduction, AM fungi-enabled ammonium assimilation conserved cellular resources, redirecting energy toward chlorophyll biosynthesis and photosynthetic output. Such metabolic flexibility underscores AM fungi’s ability to tailor N source utilization to environmental availability, favoring organic N forms like glycine under nutrient-limited condition.

The symbiosis between AM fungi and *Eucalyptus* created a nutrient acquisition feedback loop which N assimilation efficiency reinforced P utilization. Elevated GS activity in mycorrhizal roots increased demand for P-rich ATP, driving hyphal P scavenging via the AM pathway. Paradoxically, high N-P co-fertilization disrupted this synergy, excessive P suppressed AM fungi colonization, reducing NR and GOGAT activities despite nutrient abundance (**Figure 4**). This aligns with the trade-off balance model, where AM fungi benefits diminish when soil P exceeds host demand, destabilizing the symbiosis (Smith et al., 2011; Bennett and Groten, 2022). Crucially, organic N inputs sustained the synergy by stimulating phosphatase-mediated P mineralization, whereas synthetic fertilization disrupted it (Manzoor et al., 2022). These findings reveal that AM fungi-mediated nutrient synergy thrives under moderate, organically balanced fertilization but collapses under inorganic nutrient overload, highlighting the importance of AM fungi-driven stoichiometric regulation in sustainable nutrient management.

### 4.4 Effects of AM fungi on plant genes expression related to N transport under different N and P conditions

AM fungi significantly upregulated key N transporter genes in *Eucalyptus* roots under nutrient-limited conditions. Mycorrhizal seedlings exhibited elevated expression of nitrate transporters (*EgNPF4.5*, *EgNPF6.3*, *EgNPF8.1*) and organic N transporters (*EgAAP3*, *EgProt2*), compared to NM treatments (**Figure 5**), mirroring results observed in AM fungi-colonized maize and sorghum (Xie et al., 2022). This transcriptional reprogramming enables efficient N scavenging in low-fertility soils by activating dual acquisition pathways: direct nitrate uptake via NPF transporters and organic N assimilation through transport mediated by *AAP3* and *Prot2*. Notably, P limitation amplified this response, as P starvation signals cross-regulate N transporter expression, positioning AM fungi as critical enhancers of N capture under co-limiting conditions (Zhao et al., 2021).

The symbiosis shifted *Eucalyptus* N acquisition strategy toward organic sources, evidenced by upregulation of *EgAAP3* and *EgProt2*. While nitrate transporters (*EgNPF4.5*, *EgNPF6.3*, *EgNPF8.1*) were also induced, their activity likely supports secondary nitrate uptake from AM-released N pools in the peri-arbuscular space (Wang et al., 2020). Contrastingly, the downregulation of ammonium transporter *EgAMT3.1* in mycorrhizal roots suggests a metabolic trade-off, AM fungi suppress energetically costly ammonium uptake pathways while prioritizing organic N and nitrate assimilation. This aligns with findings in *Lycium barbarum*, where AM fungi colonization favors nitrate over ammonium uptake, highlighting a conserved mycorrhizal strategy to optimize N source utilization based on symbiotic efficiency (Gong et al., 2023). The coordinated and specific expression of ammonium and nitrate transporters in mycorrhizae-colonized cortical cells suggests the crucial importance of fungal N transfer in plants (Rui et al., 2022).

AM fungi orchestrated a nutrient feedback loop which P availability fine-tuned N transporter expression. High P treatments amplified *EgNPFs* and *EgAAP3* expression (**Figure 5**), indicating that P sufficiency liberates carbon resources for N transporter synthesis, while P scarcity prioritizes organic N uptake to balance stoichiometric demands. This synergy mirrors the dual role of AM fungi hyphae: (1) delivering P via the AM pathway, which reduces plant investment in root P transporters, and (2) priming N transporters to exploit organic N mineralization driven by phosphatase activity (Wang S, et al., 2025). However, excessive P disrupted the balance, downregulating AM fungi-specific ammonium transporters and demonstrating that optimal AM fungi-driven nutrient synergy occurs under moderate P levels where N-P co-regulation remains intact (Calabrese et al., 2016).

## 5 Conclusion

In conclusion, mycorrhizal *Eucalyptus* seedlings’ growth was significantly enhanced under organic N and P fertilization, compared to NM treatments. AM fungi contributed to increasing the NUE and N absorption, thus improving N contents and photosynthesis of seedlings, which in turn enhanced seedling growth. Relative expressions of genes related to organic and nitrate N transport in root of mycorrhizal seedlings were significantly higher than NM treatments, thus improving seedlings’ enzymes related to N metabolism. *Eucalyptus* showed varying preferences for absorption of various organic N sources. Under glycine treatments, mycorrhizal *Eucalyptus* seedlings’ growth was the highest promoted, whereas non-mycorrhizal *Eucalyptus* favored peptone. *Eucalyptus* growth under high level of N and P treatments were promoted markedly, regardless of organic N source, which showed N-P synergistic interaction in *Eucalyptus* seedlings. In addition, compared to NM treatments, the growth of mycorrhizal *Eucalyptus* was the most enhanced under low N and high P treatment, which has potential to reduce fertilization and improve biomass.

## Data Availability

The original contributions presented in the study are included in the article/**Supplementary Material**. Further inquiries can be directed to the corresponding author/s.
