# Supplementary material for: AM fungi modulate organic nitrogen preference of Eucalyptus to enhance seedling growth under low fertilization
Source: Front Plant Sci. 2025 Jun 20;16:1597451. doi: 10.3389/fpls.2025.1597451 (PMC12226524; doi:10.3389/fpls.2025.1597451)
Supplement: Supplementary file 1 [file DataSheet1.docx]

Supplementary Table 1. RT-qPCR primers used in this study.

| **Full gene name** | **Gene name** | **Forward primer sequence (5' - 3')** | **Reverse primer sequence (5' - 3')** |
| --- | --- | --- | --- |
| amino acid permeases | *EgAAP3* | GCTCAGCGATATAAACAACCCT | GTCCACACGGTCCCAGTT |
| lysine histidine transporter | *EgLHT1* | CGGGAGCTATCAGGTGTTCG | AACGTGATGGCCAGGAACAT |
| proline and glycine betaine transporters | *EgProt2* | GATCCCAACCATGTCTGCCA | CGTCGGATTTCCCGTCTTTG |
| ammonium transporters | *EgAMT2-1* | AGAAGTACGACCCCACGAGA | CGATCAAGCGAGAACAAGCG |
|  | *EgAMT3.1* | AGGATGAACTTTAGGGCGTG | AGGAGAGGTGGATGACGTAG |
| nitrate transporter1/ peptide transporter family | *EgNPF4.5* | ACTATCTCACCCTCTGTACCAC | CCCATAGCCAAAACGACAAAG |
|  | *EgNPF6.3* | ACTTCCGCTAACATCGTCAC | GATTGTCACACCTGTTGCTTG |
|  | *EgNPF8.1* | GACCTGCTACATTACTCCACTG | TTTAGCCCAGGAACAGAAGC |
| Ubiquitin 3 | *EgUBI3* | TCACCTACGTCTACCAGAAGG | TCCTCGAAAGCTGTAAACATGG |

Supplementary Table 2. Effects of inoculation of AM fungi, N application, P application, organic N and their interaction on various indexes of *Eucalyptus* seedlings

| Parameters | N | | P | | N*P | | N*AMF | | P*AMF | | ON*AMF | |
| --- | --- | --- | --- | --- | --- | --- | --- | --- | --- | --- | --- | --- |
|  | F | *P* | F | *P* | F | *P* | F | *P* | F | *P* | F | *P* |
| Height | 1167.92 | 0.00^**^ | 1379.98 | 0.00^**^ | 813.29 | 0.00^**^ | 813.29 | 0.00^**^ | 0.56 | 0.46^ns^ | 22.83 | 0.00^**^ |
| FW | 4390.82 | 0.00^**^ | 4168.54 | 0.00^**^ | 2826.25 | 0.00^**^ | 2826.25 | 0.00^**^ | 2.61 | 0.11^ns^ | 41.65 | 0.00^**^ |
| GD | 217.23 | 0.00^**^ | 291.80 | 0.00^**^ | 191.89 | 0.00^**^ | 191.89 | 0.00^**^ | 2.76 | 0.10^ns^ | 30.17 | 0.00^**^ |
| DW | 1698.57 | 0.00^**^ | 1916.57 | 0.00^**^ | 1047.69 | 0.00^**^ | 1047.70 | 0.00^**^ | 50.22 | 0.00^**^ | 9.01 | 0.00^**^ |
| FW | 2258.32 | 0.00^**^ | 1871.00 | 0.00^**^ | 1555.14 | 0.00^**^ | 1555.14 | 0.00^**^ | 159.63 | 0.00^**^ | 18.78 | 0.00^**^ |
| RL | 601.81 | 0.00^**^ | 2554.22 | 0.00^**^ | 292.33 | 0.00^**^ | 292.33 | 0.00^**^ | 46.45 | 0.00^**^ | 324.35 | 0.00^**^ |
| RS | 1041.76 | 0.00^**^ | 2244.84 | 0.00^**^ | 737.48 | 0.00^**^ | 737.48 | 0.00^**^ | 25.85 | 0.00^**^ | 145.39 | 0.00^**^ |
| RD | 309.02 | 0.00^**^ | 626.34 | 0.00^**^ | 11.65 | 0.001^**^ | 11.65 | 0.001^**^ | 14.44 | 0.00^**^ | 1.48 | 0.238^ns^ |
| RV | 533.52 | 0.00^**^ | 1668.78 | 0.00^**^ | 187.20 | 0.00^**^ | 187.20 | 0.00^**^ | 6.80 | 0.01^*^ | 29.75 | 0.00^**^ |
| Photo | 81.33 | 0.00^**^ | 888.37 | 0.00^**^ | 26.95 | 0.00^**^ | 26.95 | 0.00^**^ | 4.90 | 0.03^*^ | 34.13 | 0.00^**^ |
| Comd | 2792.13 | 0.00^**^ | 10023.68 | 0.00^**^ | 47.71 | 0.00^**^ | 47.71 | 0.00^**^ | 96.88 | 0.00^**^ | 180.02 | 0.00^**^ |
| Ci | 7757.31 | 0.00^**^ | 40322.87 | 0.00^**^ | 11.00 | 0.002^**^ | 11.00 | 0.002^**^ | 3607.85 | 0.00^**^ | 1349.89 | 0.00^**^ |
| Trmmol | 4960.25 | 0.00^**^ | 39306.66 | 0.00^**^ | 9.52 | 0.003^**^ | 9.52 | 0.003^**^ | 11.29 | 0.001^**^ | 523.94 | 0.00^**^ |
| Ca | 2188.99 | 0.00^**^ | 910.29 | 0.00^**^ | 297.80 | 0.00^**^ | 297.80 | 0.00^**^ | 4.81 | 0.03^*^ | 59.19 | 0.00^**^ |
| Cb | 3030.25 | 0.00^**^ | 1923.37 | 0.00^**^ | 965.65 | 0.00^**^ | 965.65 | 0.00^**^ | 20.17 | 0.00^**^ | 276.07 | 0.00^**^ |
| TC | 1256.65 | 0.00^**^ | 505.82 | 0.00^**^ | 253.42 | 0.00^**^ | 253.42 | 0.00^**^ | 3.03 | 0.09^ns^ | 68.18 | 0.00^**^ |
| TN | 179.88 | 0.00^**^ | 158.06 | 0.00^**^ | 1602.38 | 0.00^**^ | 1602.38 | 0.00^**^ | 16.43 | 0.00^**^ | 200.45 | 0.00^**^ |
| TP | 1882.78 | 0.00^**^ | 17850.87 | 0.00^**^ | 697.97 | 0.00^**^ | 697.97 | 0.00^**^ | 105.74 | 0.00^**^ | 148.40 | 0.00^**^ |
| NUE | 1766.20 | 0.00^**^ | 1722.51 | 0.00^**^ | 1568.87 | 0.00^**^ | 1568.87 | 0.00^**^ | 34.05 | 0.00^**^ | 8.70 | 0.001^**^ |
| GOGAT | 420.09 | 0.00^**^ | 589.72 | 0.00^**^ | 0.002 | 0.961^ns^ | 0.002 | 0.961^ns^ | 62.22 | 0.00^**^ | 2.22 | 0.118^ns^ |
| NR | 167.969 | 0.00^**^ | 4.16 | 0.046^*^ | 1.831 | 0.181^ns^ | 1.83 | 0.181^ns^ | 11.19 | 0.001^**^ | 9.90 | 0.00^**^ |
| GS | 146.579 | 0.00^**^ | 29.64 | 0.00^**^ | 62.62 | 0.00^**^ | 62.62 | 0.00^**^ | 10.36 | 0.002^**^ | 21.29 | 0.00^**^ |

Note: * *P*<0.05 level is significant; ** *P*<0.01 level is very significant; ns is not significant

Supplementary Figure 1. Photomicrographs of structural colonization of AM fungi in the roots of *Eucalyptus* seedlings.

a: Arbuscule structure; v: Vesicles; ih: Hypha structure.


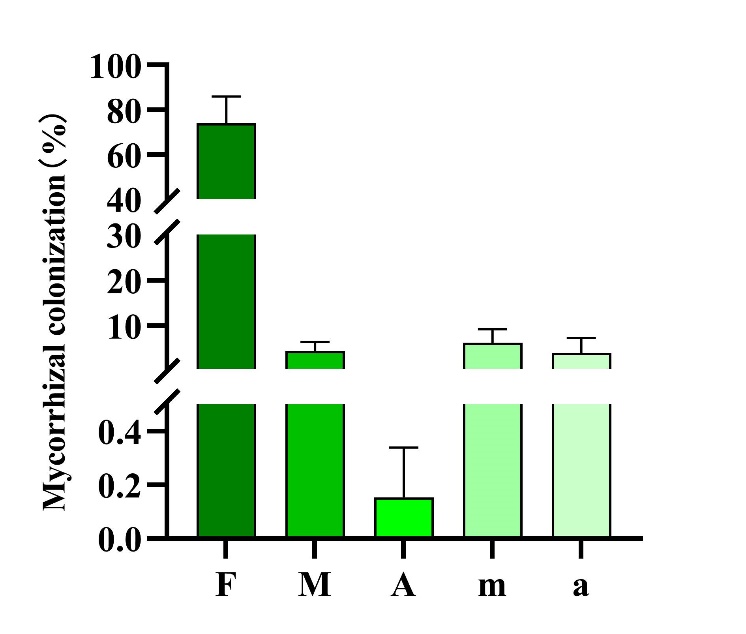


Supplementary Figure 2. The colonization of *Rhizophagus irregularis* in mycorrhizal *Eucalyptus* seedlings.

F: Percentage of colonized root fragments; M: Intensity of mycorrhizal colonization within each root fragment; A: Abundance of arbuscular throughout the root system; m: Intensity of mycorrhizal colonization throughout the root system; a: Abundance of arbuscular in colonized fragments of roots.
